# Isoflurane and Sevoflurane Induce Cognitive Impairment in Neonatal Rats by Inhibiting Neural Stem Cell Development Through Microglial Activation, Neuroinflammation, and Suppression of VEGFR2 Signaling Pathway

**DOI:** 10.1007/s12640-022-00511-9

**Published:** 2022-04-26

**Authors:** Chunlong Zuo, Junmei Ma, Yizhao Pan, Dongxu Zheng, Chunjiang Chen, Naqi Ruan, Ying Su, Haihan Nan, Qingquan Lian, Han Lin

**Affiliations:** 1 Department of Anesthesiology, The First Affiliated Hospital of AnHui Medical University, Hefei, 230022 PRC China; 2 Department of Anesthesiology, Critical Care and Pain Medicine, The Second Affiliated Hospital and Yuying Children’s Hospital of Wenzhou Medical University, Wenzhou, Zhejiang, PRC, Zhejiang Province Key Lab of Anesthesiology, The Second Affiliated Hospital and Yuying Children’s Hospital of Wenzhou Medical University, Wenzhou, 325027 PRC China; 3 Department of Anesthesiology, Ningbo Medical Center Lihuili Hospital, Ningbo, 315040 PRC China; 4 Department of Anesthesiology, The First Affiliated Hospital of Wenzhou Medical University, Shangcaicun, Wenzhou, 325000 PRC China; 5 School of Laboratory Medicine and Life Science, Wenzhou Medical University, Wenzhou, 325035 PRC China

**Keywords:** Inhaled anesthetics, Neural stem cells, Proliferation, Differentiation, Microglia, Cognitive function

## Abstract

Inhaled anesthetics are known to induce neurotoxicity in the developing brains of rodents, although the mechanisms are not well understood. The aim of this study was to elucidate the molecular mechanisms underlying anesthetics-induced neurodevelopmental toxicity by VEGF receptor 2 (VEGFR2) through the interaction between microglia and neural stem cells (NSCs) in postnatal day 7 (P7) rats. Cognitive function of P7 rats exposed to isoflurane and sevoflurane were assessed using Morris Water Maze and T maze tests. We also evaluated the expression levels of NSC biomarkers (Nestin and Sox2), microglia biomarker (CD11b or or IBA1), pro-inflammatory cytokines (IL-6 and TNF-α), and VEGFR2 using western blotting and immunohistochemistry in the brains of control and anesthesia-treated rats. We found spatial learning and working memory was impaired 2 weeks after anesthetics exposure in rats. Isoflurane induced stronger and more prolonged neurotoxicity than sevoflurane. However, cognitive functions were recovered 6 weeks after anesthesia. Isoflurane and sevoflurane decreased the levels of Nestin, Sox2, and p-VEGFR2, activated microglia, decreased the number of NSCs and reduced neurogenesis and the proliferation of NSCs, and increased the levels of IL-6, TNF-α, and CD11b. Our results suggested that isoflurane and sevoflurane induced cognitive impairment in rats by inhibiting NSC development and neurogenesis via microglial activation, neuroinflammation, and suppression of VEGFR2 signaling pathway.

## Introduction

Recent studies have demonstrated that exposure of the developing brain to anesthetics is associated with neurobehavioral abnormalities including cognitive impairments. The result from less than 1 h of general anesthesia in early infancy provides no evidence of neurocognitive deficits at age 2 or 5 years compared with awake-regional anesthesia (Davidson et al. 2016; McCann et al. 2019). However, some clinical studies of longer or repeated exposures have linked childhood anesthesia to subsequent language impairment, cognitive abnormalities, and learning disabilities (Warner et al. 2018; Wilder et al. 2009). Therefore, there is significant concern that anesthesia exposure in early infancy may have deleterious effects on the developing brain. Persistent memory and learning impairments were observed in postnatal day 7 (P7) rats exposed to a combination of anesthetics for 6 h because of extensive neuronal apoptosis and neurodegeneration (Jevtovic-Todorovic et al. 2003). Furthermore, P7 rats exposed to 1 minimum alveolar concentration (MAC) of isoflurane for 4 h showed reduced proliferation of the neural progenitor cells and defective spatial reference memory (Stratmann et al. 2009b). The exposure of P7 rats to sevoflurane also caused short-term or long-term memory deficits. The exposure of P6 mice to 3% sevoflurane for 6 h caused learning defects and abnormal social behaviors resembling autism spectrum disorders (Satomoto et al. 2009).

The exposure to 3.4% isoflurane for 6 h inhibited proliferation of primary cultured NSCs (Chen et al. 2020) and decreased mRNA levels of *Ki67* and *Sox2* in the rat neural progenitor cells (NPCs) (Sall et al. 2009). Isoflurane suppressed self-renewal of primary NSCs in a dose- and time-dependent manner (Hou et al. 2015). Sevoflurane also inhibited the self-renewability of mouse embryonic stem cells via the GABAAR-ERK signaling pathway (Wang et al. 2016).

Neurogenesis persists throughout the life of adult mammals in the subventricular zone (SVZ) of the lateral ventricles and the subgranular zone (SGZ) of the dentate gyrus (DG) in the hippocampus (Zhao et al. 2008). NSCs can differentiate into several committed neural cell types, including neurons, astrocytes, and oligodendrocytes (Kempermann et al. 2004). The expression of doublecortin (*DCX*) gene is temporally regulated during neurogenesis, and is typically expressed during the first 2 weeks after the birth of the neurons. The expression of neuronal nuclei (*NeuN*) gene begins during the first few days of neuronal cell development and persists in the mature neurons. In the granule cells, expression of the calcium-binding protein, calretinin, is observed between the 2nd and 4th weeks of neurogenesis (Zhao et al. 2008). It has been shown that isoflurane or sevoflurane can induce inhibition of neurogenesis in the hippocampus of rodent animals (Jia et al. 2020; Li et al. 2021a).

Microglia is the main regulator of neuroinflammation and the only immune cell type that permanently resides in the central nervous system (CNS) alongside neurons (Tambuyzer et al. 2009; Tay et al. 2017). Microglial cells play a dual role in neurogenesis based on the nature and duration of brain inflammation (Ekdahl et al. 2009; Whitney et al. 2009). Activated microglial cells inhibit neurogenesis via pro-inflammatory cytokines, such as TNF-α and IL-6 (Cacci et al. 2008; Monje et al. 2003; Ryan and Nolan 2016). Foreign antigens or changes in the brain homeostasis activate microglial cells, which subsequently promote neuroinflammation and suppress hippocampal neurogenesis (Walton et al. 2006). Furthermore, anesthetics such as isoflurane or sevoflurane promote secretion of inflammatory cytokines from the microglia (Zhang et al. 2013). Both in vivo and in vitro studies have demonstrated that activated microglial cells inhibit neurogenesis via neuroinflammation (Monje et al. 2003). However, little is known about the interaction between microglia and NSCs and anesthetics-induced neurodevelopmental toxicity or cognitive dysfunction.

VEGFR2 (VEGF receptor 2) is the main receptor that regulates VEGF-A-mediated trophic effects in the CNS (Licht et al. 2011). VEGFR2 signaling promotes proliferation, migration, and differentiation of the NSCs (Jin et al. 2002; Sun et al. 2006). VEGFR2 is significantly expressed in the NPCs (Ogunshola et al. 2002). Furthermore, learning and memory can be regulated via VEGFR-2 (Deyama et al. 2020). In a diabetic foot ulcer rat model, Nrf2 overexpression can increase VEGFR2 phosphorylation, promotes proliferation and angiopoiesis in endothelial progenitor cells by reducing levels of inflammatory cytokines such as IL-6 and TNF-α (Li et al. 2018). Besides, in an ischemic hind limb model, ischemic wound healing may be associated with enhanced levels of phosphorylated VEGF receptors through reduction of inflammatory response (Li et al. 2021b). Thus, we postulated that VEGFR2 phosphorylation might be inhibited by neuroimflammation.

We therefore set out to determine whether isoflurane and sevoflurane can induce neurodevelopmental toxicity and cognitive dysfunction through the interaction between microglia and NSCs and whether its effects are associated with the changes of VEGFR2 phosphorylation.

## Material and Methods

### Animals

All animal experimental protocols were approved by the Animal Care and Use Committee of Wenzhou Medical University (Wenzhou, Zhejiang, China), and all procedures were performed following the National Institutes of Health (NIH, Bethesda, MD, USA) guidelines of animal care. The P7 rats were housed under a 12:12 h light–dark cycle at 22–24 ℃ ambient temperature with their parents. They were randomly assigned to the neonatal rats control (CON), neonatal rats anesthetized with isoflurane (ISO), and sevoflurane (SEV) groups.

### Anesthesia in Rats

The P7 rats were anesthetized at 1 minimum alveolar concentration (MAC) as determined by tail clamping. The 1 MAC of isoflurane or sevoflurane was determined to be 1.1% or 2.0% concentrations, respectively. The ISO or SEV group was flushed continuously with isoflurane or sevoflurane and 30% oxygen for 4 h. The CON group received 30% oxygen for 4 h at the identical condition. During anesthesia, neonatal rats breathed spontaneously. The temperature of the chamber floor was kept at 37 ℃ and was covered with soda lime. The concentration of isoflurane was continuously monitored using a gas analyzer (ARYM-0054 Vamos, Dräger, Germany). When the rats were exposed to anesthetics in chambers, respiratory rate and invasive arterial blood pressure were monitored and blood was sampled for blood gas analysis. Anesthesia was terminated by discontinuing isoflurane or sevoflurane. Then the rats were kept in a chamber containing 30% oxygen until the return of righting reflex. Subsequently, they were returned to their home cages with their parents.

### BrdU Administration

The rats were administered bromodeoxyuridine (BrdU; Sigma, St. Louis, MO; 20 mg/ml) at 100 mg/kg/d for 4 d (intraperitoneally; dissolved in PBS) after isoflurane or sevoflurane exposure.

BrdU has been a principal marker for mitotic cells in studies of the neurogenesis (Gratzner 1982). This method labels freshly divided neural stem/progenitor cells and benefits from its long-term retention in divided cells and its passage to their daughter cells (Schmuck et al. 2014). This feature can be used to trace the cell lineage and cell survival.

### Hemodynamic Monitoring and Blood Gas Analysis

Blood pressure and blood gases were measured in a separate cohort (n = 8/group) as previously described to confirm whether such an anesthesia regimen affects cardiorespiratory function. Briefly, arterial blood was sampled in the ISO or SEV rats via a 24-gauge arterial puncture needle (IntroCan®-W, Braun Medical Inc., Bethlehem, PA, USA) through the abdominal aorta using a dissecting microscope (PS100, Nikon, Tokyo, Japan). The mean arterial blood pressure (MAP) was measured by an anesthesia monitor (M3046, Philips Medical System, Boeblingen, Germany). The blood sample (0.2-0.03 ml) was immediately analyzed to determine pH, arterial oxygen, and carbon dioxide using the blood gas analyzer (GEM Premier 3000, Bedford, MA, USA).

### Open-field Test

The open-field test (OFT) was done at 2 weeks after the isoflurane or sevoflurane exposure. The animals were brought to the experimental room 5–20 min before testing to allow habituation. Each animal was placed in a corner square of the open field and faced the corner. Subsequently, each animal was observed for 5 min each time for three times. After 5 min, the animal was removed. Then the movement distance, time spent, and number of entries to the central region of the animal were recorded.

### Morris Water Maze (MWM)

The MWM was done at 2 weeks (P21) and 6 weeks (P49) after the isoflurane or sevoflurane exposure. Before the trials, all rats were placed in the water of the swimming arena with a 6 cm diameter platform submerged 0.5–1 cm above the surface of the water on day 0. Each rat was allowed to swim for 120 s to locate the platform. The rats that had vision problems or did not swim were removed from the arena and excluded from further experiments. Subsequently, spatial acquisition trials were conducted, where the platform was submerged 0.5–1 cm below the surface of the water. The animals underwent four trials each day in the pool at four different starting positions facing the tank wall. A time limit of 2-min per trial allowed for rats to find the platform within a 30-min inter-trial interval. The animals not finding the platform within the time allotted were guided onto the platform for 15 s. The swim speed, distance (path length), and time (escape latency) in finding the platform were calculated from the recorded videos using the MWM software (SLY-WMS Morris water maze, Shuolinyuan, Beijing, China). On day 6, a probe trial was performed, during which the platform was removed, and animals were placed in a novel start position 180° from the original platform position to swim freely for 30 s. The percentage of time spent in the target quadrant and the time on platform–site crossovers were recorded.

### T Maze

The T maze was done at 2 weeks and 6 weeks after the isoflurane or sevoflurane exposure. Before trials, each animal was maintained at 90%-95% of its free-feeding body weight. Then the animals were placed into a T maze for 3 min each day for successive four days. For the forced trials, the reward food (milk) was placed in the food well at one goal arm, and the other goal arm door was blocked. After opening the central partition at the start arm, each animal, which was placed in the start area, ran for the reward food. When the animal consumed all the reward food, the animal was returned to the start arm and the start arm door was then closed. Then, for rewarded alternation trials, each animal was stood in the start area facing away from the goal arms for 15 s. After 15 s, the central partition and the doors of the goal arms were removed. The animal was allowed to choose between the two open goal arms. The time was allowed to consume the reward if correct. If the animal chose incorrectly the animal was removed after a time period equivalent to the time normally used to consume the reward to ensure that it had definitely discovered that the sample well was empty. As with rewarded alternation, each trial took no more than 2 min. The two trials (forced trials and rewarded alternation trials) were alternated, and the reward food at one goal arm was random, and the numbers of reward food were equal for two goal arms. The numbers of correct alternations were recorded for each animal.

### Western Blot

Eight animals from each group were deeply anesthetized with 5% chloral hydrate and transcardially perfused with normal saline 5 days after anesthetics exposure. The brains were quickly removed. The hippocampus tissue was homogenized to a mixture composed of RIPA lysis buffer, phosphatase, and protease inhibitors, and incubated for 30 min on ice. The lysate was then sonicated and centrifuged at 12,000 rpm for 30 min at 4 ℃. The protein samples were quantitated using bicinchoninic acid (BCA) protein assay kit (Thermo Scientific, Eugene, OR, USA) and the concentrations were measured using a spectrophotometer (Multiskan MK3, Thermo scientific). Subsequently, the samples were admixed with 5 × sample buffer, equalized using double-distilled H_2_O, and heated for 5 min at 100 ℃. An equal amount of protein from each sample was separated using sodium dodecyl sulfate–polyacrylamide gel electrophoresis (SDS-PAGE) on a 10% gel and then electrophoretically transferred to a polyvinylidene fluoride (PVDF) membrane (Bio-Rad Laboratories Inc., Hercules, CA, USA). The blots were blocked with 10% skim milk in Tris-buffered saline and Tween 20 (0.1%) (TBST) for 2 h at room temperature (RT) and incubated at 4 ℃ overnight with mouse Nestin antibody (1:500, MAB353, Millipore), rabbit Sox2 antibody (1:1000, ab97959, Abcam), rabbit cd11b antibody (1:500, DF6476, Affinity), rabbit IL-6 antibody (1:500, DF6087, Affinity), rabbit IL-1β (1:500, AF5103, Affinity), rabbit TNF-α (1:500, AF7014, Affinity), rabbit VEGFR2 (1:500, AF6281, Affinity), rabbit p-VEGFR2 (1:500, AF8022, Affinity), or rabbit β-actin antibody (1:1000, AP0060, Bioworld Technology). After incubation with the primary antibody, the blots were incubated for 2 h at RT with secondary antibodies [goat anti-rabbit antibodies (1:5000, 111–035-003), and goat anti-mouse antibodies (115–035-003), Jackson]. Between steps, the blots were washed with TBST. The blots were visualized using ECL western blot detection system (ImageQuant LAS 4000 Mini). The bands were analyzed by Quantity One software version 4.6.2 (Bio-Rad Laboratories Inc.).

### Immunohistochemistry

Six animals from each group were deeply anesthetized with 5% chloral hydrate and transcardially perfused with normal saline with simultaneous exsanguination from the right atrium, and then with 4% paraformaldehyde in 0.1 M phosphate buffer at a pH of 7.4, 5 days after anesthesia exposure (P12) and 24 h after the MWM (P28 and P56). The brains were removed and kept in 4% paraformaldehyde at 4 ℃ overnight. Subsequently, the brains were consecutively dipped into 70%, 85%, 95%, and 100% ethanol. Serial coronal 5 µm sections were cut in paraffin blocks using a microtome (Leica R2016), and at least three slides from each animal were used for staining. Briefly, sections were deparaffinized with xylene, rehydrated with a series of graded ethanol, and washed in distilled water and then in PBS. The antigen retrieval was performed incubating in 10 mM sodium citrate buffer at a pH of 6.0 for 20 min in a microwave oven at 100 ℃. The sections were blocked in 5% BSA in PBS for 30 min and were then incubated with primary antibodies (mouse anti-Nestin, 1:100, Millipore; rabbit anti-Sox2, 1:100, Millipore; rabbit anti-GFAP, 1:500, Dako; sheep anti-BrdU, 1:500, Abcam; rabbit anti-DCX, 1:400, Abcam; rabbit anti-NeuN, 1:200, Abcam; rabbit anti-IBA1, 1:400, Wako) overnight at 4 ℃. Subsequently, the sections were washed at RT, incubated in fluorophore-conjugated secondary antibodies (donkey anti-sheep, 1:500, ab150177, Abcam; donkey anti-mouse, 1:500, ab150112, Abcam; donkey anti-rabbit, 1:500, ab150068, Abcam; donkey anti-rabbit, 1:500, ab150073, Abcam) for 50 min, and then washed. The tissue sections were then incubated in DAPI (1:1000, Sigma) and then washed with PBS. Finally, the sections were mounted, viewed and quantitated using a microscope (Nikon E100) captured with 200 × magnification. The NSC proliferation (BrdU/Nestin, BrdU/Sox2), numbers (GFAP/Sox2) and differentiation (BrdU/DCX, BrdU/NeuN) of the NSC, microglia activation (IBA1^+^), and the number of neuronal nuclei in the DG (DAPI) were examined. Five random sections per brain were immunostained, and photomicrographs were captured at × 200 magnification. The average of positive cell numbers of five random sections represented one brain. The total number of cells in the hippocampus was assessed by counting the neuronal nuclei stained with DAPI. All counting by investigators was blinded. All quantifications were determined by calculating the percentage of IBA1^+^ or double-labeled positive cells to the total cells of cross-sectional hippocampus area in five random sections per rat using Image-Pro Plus software version 6.0. A ratio was calculated and results presented as levels of expression (positive/total cells). The methods were analyzed as previously described (Shen et al. 2013).

### Statistical Analysis

All the data were expressed as mean ± SEM except those data derived from the probe trials of MWM that were expressed as median and interquartile range. The data of spatial acquisition trials were analyzed by a two-way ANOVA with repeated measures (isoflurane or sevoflurane exposure as one factor between subjects and day as a repeated measure factor) followed with the LSD post hoc test comparison. MANOVA was used to test the main effects for a group at each time point. The data of the probe trial were analyzed using a one-way ANOVA (isoflurane or sevoflurane as one variable). The level of protein expression and the levels of markers in each group were analyzed with one-way ANOVA followed by the LSD post hoc test. A *p*-value less than 0.05 was considered statistically significant. The SPSS software (SPSS for Windows, version 24.0, SPSS, Chicago, IL, USA) was used to analyze the data.

## Results

### Exposure of Neonatal Rats to Isoflurane and Sevoflurane Does Not Cause Cardiorespiratory Distress

We analyzed the effects of isoflurane or sevoflurane on cardiorespiratory functions by exposing neonatal rats (n = 8 each per group) to 1.1% isoflurane (ISO) or 2% sevoflurane (SEV) for 4 h. However, we did not observe any significant differences in the respiratory rates between the control (CON), ISO and SEV groups of neonatal rats (data not shown). Arterial blood gas analysis demonstrated that partial pressure of oxygen (PaO_2_), partial pressure of carbon dioxide (PaCO_2_), and pH were all similar and within the normal range for the CON, ISO, and SEV groups of neonatal rats; moreover, mean arterial pressure (MAP) was similar for the CON, ISO, and SEV groups of neonatal rats (Table 1).

**Table 1:** Results of MAP and blood gas analysis among the groups (Mean ± SEM, n = 8)

Parameters		CON	ISO	SEV
pH		7.39 ± 0.02	7.37 ± 0.02	7.39 ± 0.02
PaCO_2_	(mmHg)	36.8 ± 1.9	40.1 ± 1.7	39.5 ± 1.0
PaO_2_	(mmHg)	166.3 ± 4.4	159.5 ± 2.8	162.88 ± 2.4
MAP	(mmHg)	51 ± 1	49 ± 1	50 ± 1

Measurement of the MAP and blood gas value of rats at 4 h after anesthesia among groups. pH, p = 0.706; one-way ANOVA (F = 0.354; df = 2); PaCO_2_, p = 0.308; one-way ANOVA (F = 1.247, df = 2); PaO_2_, p = 0.370; one-way ANOVA (F = 1.042, df = 2); MAP, p = 0.318; one-way ANOVA (F = 1.211; df = 2)

*ISO* Rats anesthetized with isoflurane group, *SEV* Rats anesthetized with sevoflurane group

### Inhalation of Isoflurane and Sevoflurane Does Not Affect Activity and Behavior of Rats

**Fig. 1:**
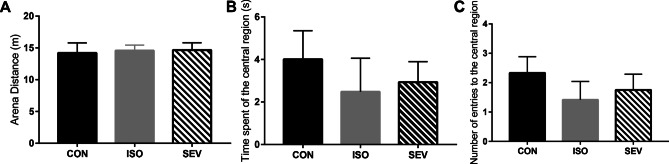
Activity and behavior of rats in the open field. The behavior of rats in each group (n = 12) was detected within 5 min. **A, B, C.** The results showed that the total distance traveled within the arena, time spent and number of entries to the central region of the open-field arena did not significantly differ in each group

We then assessed the effects of isoflurane and sevoflurane on the general activity and behavior of rats (n = 12 each per group). The open-field test results at 2 weeks after exposure to the anesthetics showed that the total distance traveled within the test arena, time spent, and number of entries to the central region of the open-field arena were similar for the CON, ISO, and SEV groups of rats (Fig. 1). These data suggested that the inhaled anesthetics did not affect activity and behavior of rats.

### Inhalation of Isoflurane or Sevoflurane Impairs Short-term Learning and Memory of Rats

**Fig. 2:**
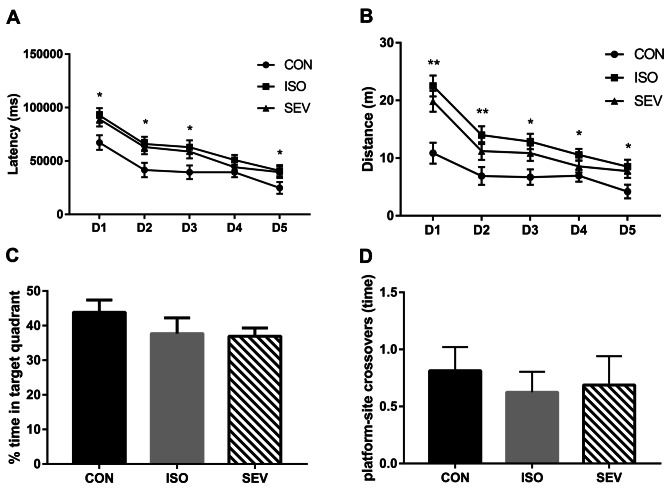
Spatial learning and memory of rats (n = 15) in each group were assessed 2 weeks after isoflurane and sevoflurane anesthesia. Neonatal rats received 4 h of isoflurane or sevoflurane and underwent assessment of spatial learning and memory in the MWM. Mice navigated to the hidden platform in place trials. During a probe trial, memory retention was assessed 24 h after the place trials. **A.** Time to reach the platform (escape latency) was recorded in the MWM. **B.** Escape distance was recorded in the MWM. On days 1, 2 and 3, escape latency or distance of the MWM increased in ISO or SEV group compared to those in CON group. On the day 4, escape distance increased in the ISO group compared to that in the CON group. On the day 5, although escape latency in the ISO or SEV group tended to increase compared to that in the CON group, differences were significant only between the ISO and CON group. However, the escape distance still increased in the ISO or SEV group compared to that in the CON group. **C.** The percentage of time spent in the target quadrant was recorded in the MWM. **D.** The time of platform–site crossovers was recorded in the MWM. During the probe trial, although the ISO or SEV group tended to take less time at the platform–site crossovers and spend less time in the target quadrant compared to the CON group, differences were not significant among all groups. **p < 0.01, *p < 0.05

We performed Morris water maze (MWM) tests to determine the effects of isoflurane and sevoflurane on the learning and memory of rats. The ISO and SEV group rats (n = 15 each per group) showed higher MWM escape latencies and distances compared to the CON group rats (n = 15) on days 1, 2, and 3 [Fig. 2A,B; MWM escape latency: *p* = 0.011 (ISO vs. CON); *p* = 0.028 (SEV vs. CON); MANOVA (F = 4.102, df = 2) for day 1; *p* = 0.015 (ISO vs. CON); *p* = 0.030 (SEV vs. CON); MANOVA (F = 3.882, df = 2) for day 2; *p* = 0.013 (ISO vs. CON); *p* = 0.038 (SEV vs. CON); MANOVA (F = 3.867, df = 2) for day 3; MWM escape distance: *p* = 0.000 (ISO vs. CON); *p* = 0.001 (SEV vs. CON); MANOVA (F = 11.339, df = 2) for day 1; *p* = 0.002 (ISO vs. CON); *p* = 0.053 (SEV vs. CON); MANOVA (F = 5.408, df = 2) for day 2; *p* = 0.002 (ISO vs. CON); *p* = 0.033 (SEV vs. CON); MANOVA (F = 5.581, df = 2) for day 3]. The ISO group (n = 15) showed significantly increased escape distance compared to the CON group (n = 15) on day 4 [Fig. 2A,B; *p* = 0.017; MANOVA (F = 3.110, df = 2)].

**Fig. 3:**
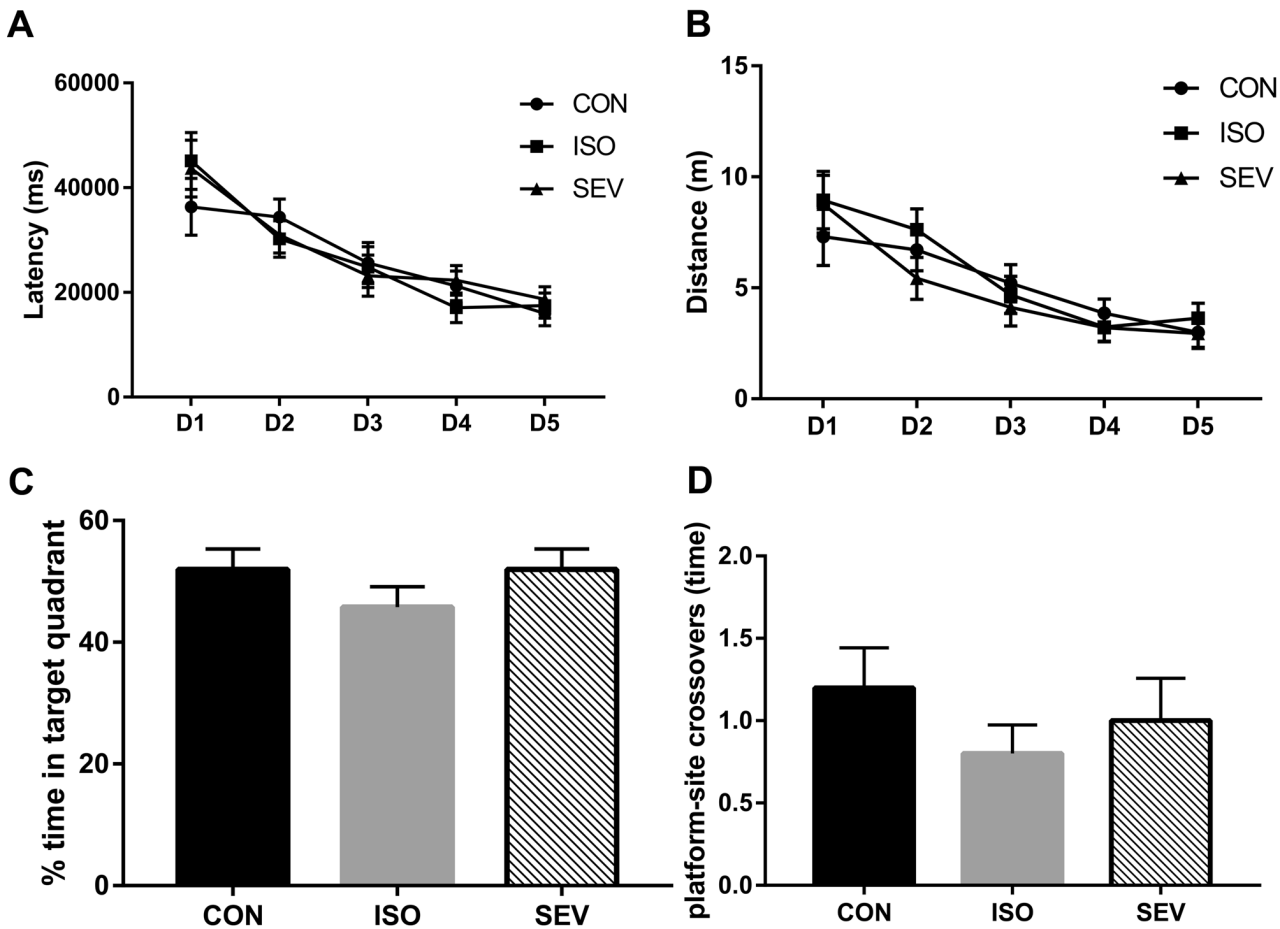
Spatial learning and memory of rats (n = 15) in each group were assessed 6 weeks after isoflurane and sevoflurane anesthesia. **A.** Time to reach the platform (escape latency) was recorded in the MWM. **B.** Escape distance was recorded in the MWM. **C.** The percentage of time spent in the target quadrant was recorded in the MWM. **D.** The time of platform–site crossovers was recorded in the MWM. The results of the MWM 6 weeks after isoflurane or sevoflurane exposure found that the performance did not differ significantly

The MWM escape latencies of both ISO and SEV groups of rats (n = 15) were higher than the CON group (n = 15) on day 5, but the differences were significant only between the ISO and CON groups [Fig. 2A,B; *p* = 0.045; MANOVA (F = 2.658, df = 2)]. However, escape distances of both the ISO and SEV groups of rats were significantly higher than the CON group rats on day 5 [Fig. 2B; *p* = 0.013 (ISO vs. CON); *p* = 0.039 (SEV vs. CON); MANOVA (F = 3.829, df = 2)]. During the probe trial on day 6, time spent at the platform–site crossovers and percentage time in the target quadrant was lower for the rats from the ISO and SEV groups compared to the CON group, however, the differences were not statistically significant [Fig. 2c,d; one-way ANOVA (F = 1.627; df = 2), p = 0.209 for the percentage of time in the target quadrant; one-way ANOVA (F = 0.204; df = 2), p = 0.816 for platform–site crossovers]. The MWM test results6 weeks after exposure to the anesthetics did not show any significant differences between the CON, ISO, and SEV groups of rats (Fig. 3A–D).

These results suggested that cognitive function was impaired in the rats 2 weeks after exposure to isoflurane or sevoflurane. However, cognitive function was restored 6 weeks after exposure to the anesthetics.

### Exposure to Isoflurane and Sevoflurane Impairs Working Memory of Rats

**Fig. 4:**
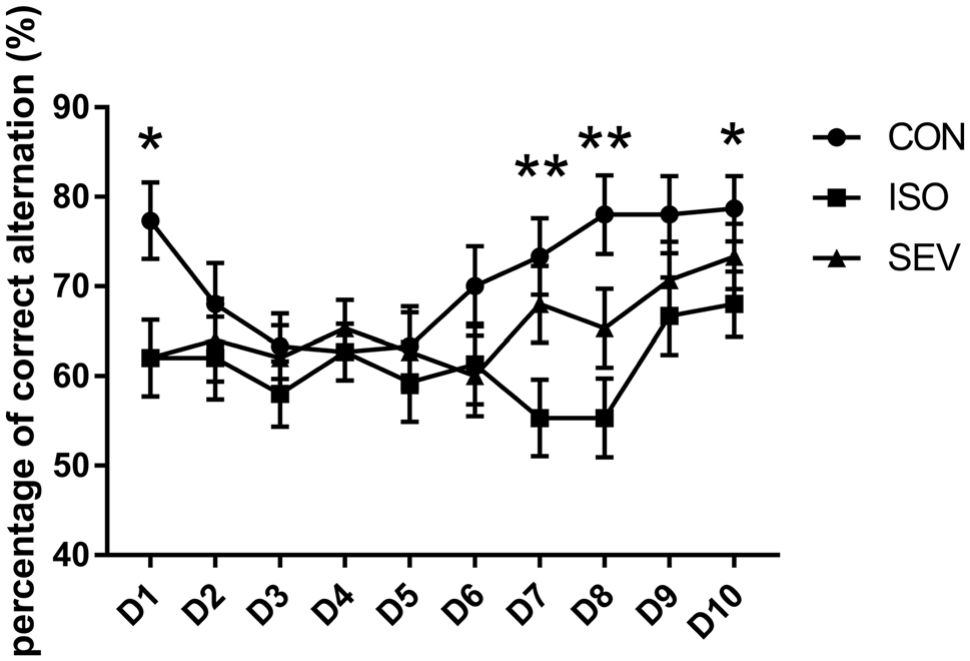
Working memory of rats (n = 15) in each group were assessed 2 weeks or 6 weeks after isoflurane and sevoflurane anesthesia. The percentages of correct alternations were significantly lower on days 1 and 8 for the ISO and SEV group rats compared to the CON group rats [Fig. 4; day 1: p = 0.015 (ISO vs. CON); p = 0.015 (SEV vs. CON); MANOVA (F = 4.266, df = 2); day 8: p = 0.001 (ISO vs. CON); p = 0.048 (SEV vs. CON); MANOVA (F = 6.671, df = 2)]. The percentage of correct alternations on days 7 and 10 was significantly lower for the ISO group compared to the CON group [Fig. 4; day 7: p = 0.005; MANOVA (F = 4.664, df = 2); day 10: p = 0.044; MANOVA (F = 2.147, df = 2)]. And the percentage of correct alternations on days 7 was significantly lower for the ISO group compared to the SEV group [Fig. 4; day 7: p = 0.043; MANOVA (F = 4.664, df = 2)]. Data are mean ± SEM. **p < 0.01, *p < 0.05

**Fig. 5:**
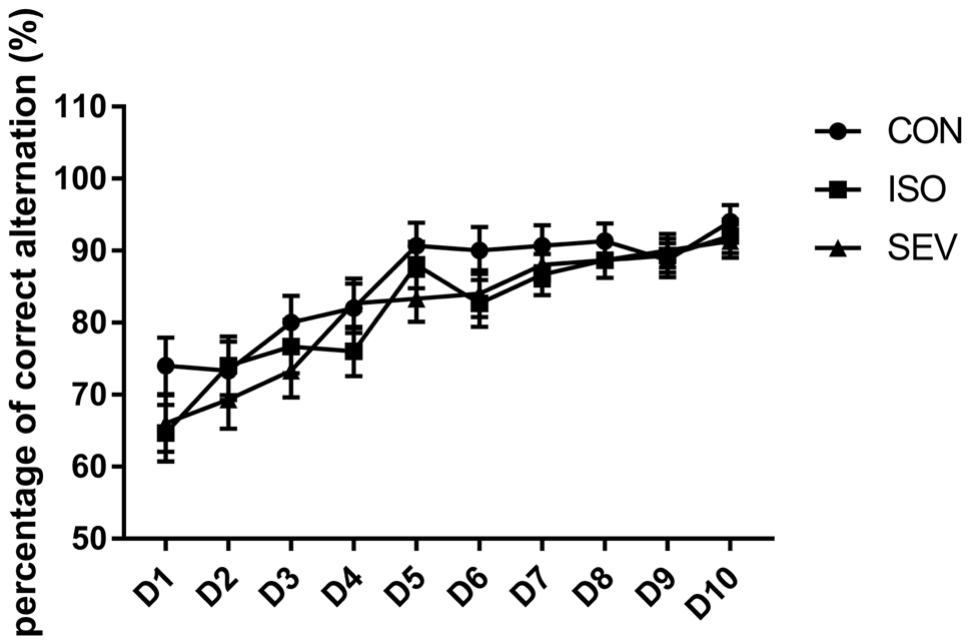
The results showed that the percentage of the correct alternation did not differ significantly between groups 6 weeks after isoflurane and sevoflurane anesthesia. Data are mean ± SEM. **p < 0.01, *p < 0.05

We then analyzed the working memory of rats (n = 15 each per group) using the T maze test 2 weeks after exposure to the anesthetics. The percentages of correct alternations were significantly lower on days 1 and 8 for the ISO and SEV group rats compared to the CON group rats [Fig. 4; day 1: *p* = 0.015 (ISO vs. CON); *p* = 0.015 (SEV vs. CON); MANOVA (F = 4.266, df = 2); day 8: *p* = 0.001 (ISO vs. CON); *p* = 0.048 (SEV vs. CON); MANOVA (F = 6.671, df = 2)]. The percentage of correct alternations on days 7 and 10 was significantly lower for the ISO group compared to the CON group [Fig. 4; day 7: *p* = 0.005; MANOVA (F = 4.664, df = 2); day 10: *p* = 0.044; MANOVA (F = 2.147, df = 2)]. And the percentage of correct alternations on days 7 was significantly lower for the ISO group compared to the SEV group [Fig. 4; day 7: *p* = 0.043; MANOVA (F = 4.664, df = 2)]. Moreover, the percentage of correct alternations was lower for the SEV group of rats compared to the CON group rats on days 7 and 10, but the differences were statistically insignificant (Fig. 4). These results showed that the working memory of rats was impaired 2 weeks after isoflurane or sevoflurane exposure. However, the percentage of correct alternations 6 weeks after exposure to the anesthetics was similar for all three groups (Fig. 5). This suggested that working memory was restored 6 weeks after exposure to anesthetics.

### Exposure to Isoflurane or Sevoflurane Inhibits VEGFR2 Signaling and Increases Neuroinflammation by Activating Microglia

**Fig. 6:**
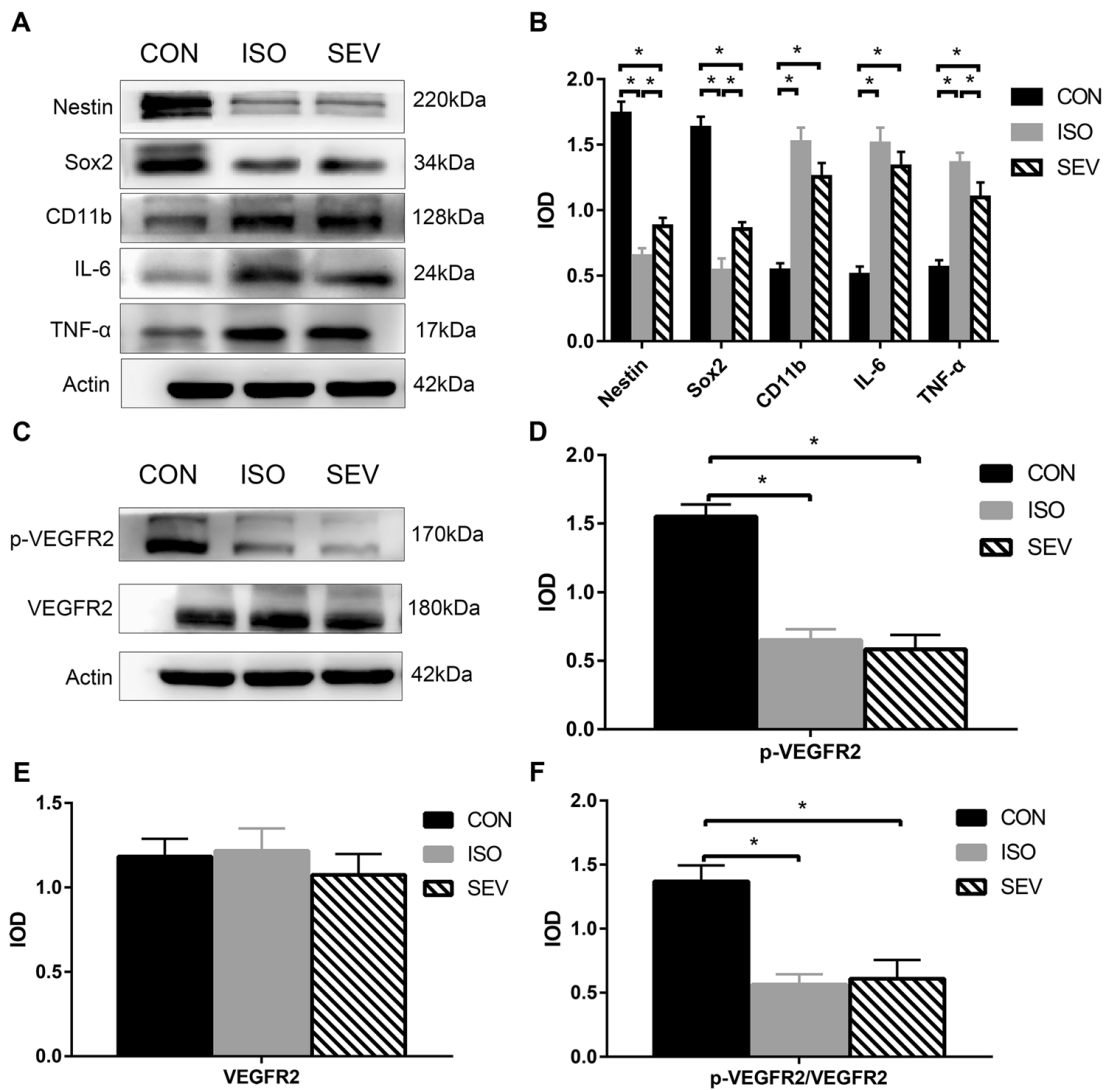
The protein expression levels of Nestin, Sox2, CD11b, IL-6, TNF-α and p-VEGFR2/VEGFR2 in the hippocampus (n = 8 each per group) were detected 5 days after isoflurane and sevoflurane anesthesia. **A, B.** The results revealed that isoflurane (*p* = 0.000, for Nestin; *p* = 0.000, for Sox2) and sevoflurane (*p* = 0.000, for Nestin; *p* = 0.000, for Sox2) inhibited the expression of Nestin and Sox2. Isoflurane inhibited the expression of Nestin or Sox2 compared to that of sevoflurane (*p* = 0.040, for Nestin; *p* = 0.010, for Sox2). Furthermore, isoflurane (*p* = 0.000, for IL-6; *p* = 0.000, for TNF-α; *p* = 0.000, for CD11b) and sevoflurane (*p* = 0.000, for IL-6; *p* = 0.000, for TNF-α; *p* = 0.000, for CD11b) increased the levels of IL-6, TNF-α and the microglial marker CD11b in hippocampus. **C-F.** Isoflurane (*p* = 0.000, for p-VEGFR2; *p* = 0.000, for p-VEGFR2/VEGFR2) and sevoflurane (*p* = 0.000, for p-VEGFR2; *p* = 0.000, for p-VEGFR2/VEGFR2) decreased the level of p-VEGFR2 and the ratio of p-VEGFR2/VEGFR2. Data are mean ± SEM. **p < 0.01, *p < 0.05

The levels of IL-6, TNF-α, and CD11b (microglial marker) were significantly higher in the hippocampus of ISO (Fig. 6A,B; *p* = 0.000 for IL-6; *p* = 0.000 for TNF-α; *p* = 0.000 for CD11b) and SEV (Fig. 6A,B; *p* = 0.000 for IL-6; *p* = 0.000 for TNF-α; *p* = 0.000 for CD11b) in groups of rats. This suggested increased neuroinflammation due to microglial activation after exposure to anesthetics. Furthermore, in comparison with the CON group, the expression levels of *Nestin*, *Sox2,* and p-VEGFR2 as well as the ratio of p-VEGFR2/VEGFR2 were significantly reduced in the hippocampus of rats belonging to the ISO group (Fig. 6A–F; *p* = 0.000 for Nestin; *p* = 0.000 for Sox2; *p* = 0.000 for p-VEGFR2; *p* = 0.000 for p-VEGFR2/VEGFR2) and SEV group (Fig. 6A–F, p = 0.000 for Nestin; *p* = 0.000 for Sox2; *p* = 0.000 for p-VEGFR2; *p* = 0.000 for p-VEGFR2/VEGFR2). These results suggested reduced activity of VEGFR2 in the hippocampus of rats after exposure to the anesthetics. Furthermore, expression levels of Nestin and Sox2 mRNAs were significantly lower in the hippocampus of rats belonging to the ISO group compared to those belonging to the SEV group (Fig. 6A,B; *p* = 0.040 for Nestin; *p* = 0.010 for Sox2). This suggested inhibition of the downstream VEGF/VEGFR2 signaling pathway.

### Isoflurane and Sevoflurane Exposure Activates Microglia and Inhibits Proliferation and Differentiation of NSCs in the Hippocampus of Rats

**Fig. 7:**
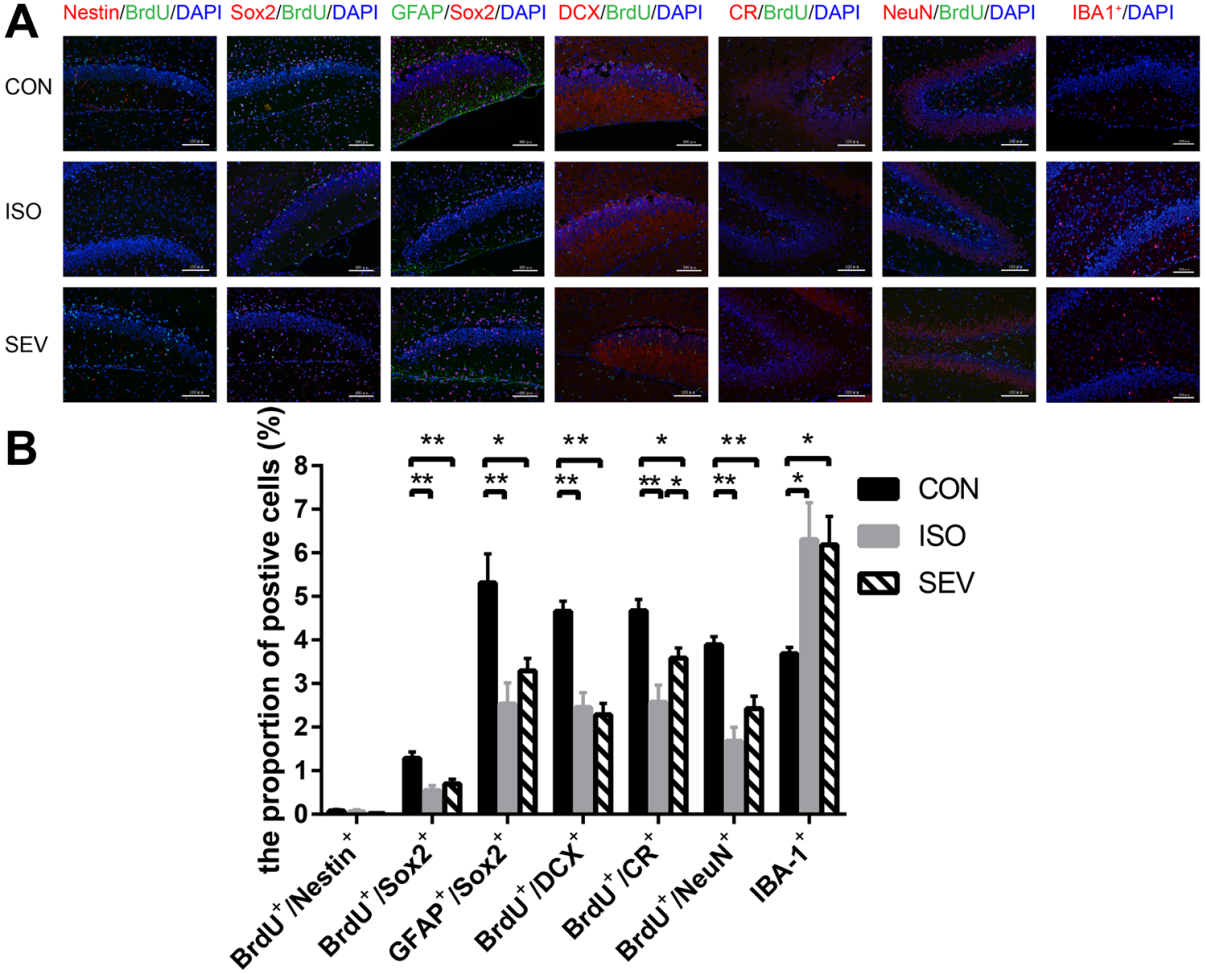
Neurodevelopmental markers for NSC in the hippocampus were stained 5 days after isoflurane and sevoflurane anesthesia. **A, B.** The results revealed that the level of Nestin^+^/BrdU^+^ co-labeling decreased in the ISO or SEV group compared to that in the CON group, but there was no significant difference among these groups. However, the level of Sox2^+^/BrdU^+^ co-labeling decreased in the ISO (*p* = 0.001, for Sox2^+^/BrdU^+^) or the SEV (*p* = 0.004, for Sox2^+^/BrdU^+^) group compared with that in the CON group. And the level of Sox2^+^/GFAP^+^ co-labeling decreased in the ISO (*p* = 0.001, for Sox2^+^/GFAP^+^) or the SEV (*p* = 0.013, for Sox2^+^/GFAP^+^) group compared with that in the CON group. Then the levels of DCX^+^/BrdU^+^, CR^+^/BrdU^+^ and NeuN^+^/BrdU^+^ co-labeling decreased in the ISO (*p* = 0.000, for DCX + /BrdU + ; *p* = 0.000, for CR^+^/BrdU^+^; *p* = 0.000, for NeuN^+^/BrdU^+^) or the SEV (*p* = 0.000, for DCX^+^/BrdU^+^; *p* = 0.023, for CR^+^/BrdU^+^; *p* = 0.002, for NeuN^+^/BrdU^+^) group compared with that in the CON group. The level of CR^+^/BrdU^+^ co-labeling decreased in the ISO group compared with that in the SEV group (*p* = 0.032). Furthermore, the numbers of IBA-1^+^cells increased in the ISO (*p* = 0.010) or the SEV (*p* = 0.013) group compared with that in the CON group. Data are mean ± SEM. **p < 0.01, *p < 0.05. Images were captured with 200 × magnification

We then performed Immunohistochemical (IHC) analysis to assess the effects of isoflurane and sevoflurane on the proliferation and differentiation of NSCs. IHC results demonstrated that Nestin^+^/BrdU^+^ co-labeling was reduced in the ISO and SEV groups on day 5 after exposure to the anesthetics compared to the CON group, but these differences were not statistically significant. However, significant reduction in Sox2^+^/BrdU^+^ co-labeling was observed in the ISO and the SEV groups compared to the CON group [Fig. 7A,B; *p* = 0.001 (ISO vs. CON) and *p* = 0.004 (SEV vs. CON)]. This suggested that the anesthetics decreased the proliferation of NSCs. Furthermore, we observed decreased Sox2^+^/GFAP^+^ co-labeling in the ISO and SEV groups compared to the CON group [Fig. 7A,B; *p* = 0.001 (ISO vs. CON); *p* = 0.013 (SEV vs. CON)]. This showed that the anesthetics decreased the number of NSCs in the hippocampus of rats. We also observed reduced DCX^+^/BrdU^+^, CR^+^/BrdU^+^, and NeuN^+^/BrdU^+^ co-labeling in the ISO (Fig. 7A,B; *p* = 0.000 for DCX^+^/BrdU^+^; *p* = 0.000 for CR^+^/BrdU^+^; *p* = 0.000 for NeuN^+^/BrdU^+^) and SEV (Fig. 7A,B; *p* = 0.000 for DCX^+^/BrdU^+^; *p* = 0.023 for CR^+^/BrdU^+^; *p* = 0.002 for NeuN^+^/BrdU^+^) groups compared to the CON group. We also observed decreased CR^+^/BrdU^+^ co-labeling in the ISO group compared to the SEV group (Fig. 7A,B; *p* = 0.032). These results showed that the anesthetics inhibited differentiation of NSCs. We also observed significantly higher numbers of IBA-1^+^cells in the ISO and SEV groups compared to the CON group [Fig. 7A,B; *p* = 0.010 (ISO vs. CON); *p* = 0.013 (SEV vs. CON)]. IBA-1 is a marker of microglial activation (Norden et al. 2016). This showed that the anesthetics activated the microglia in the hippocampus of rats.

**Fig. 8:**
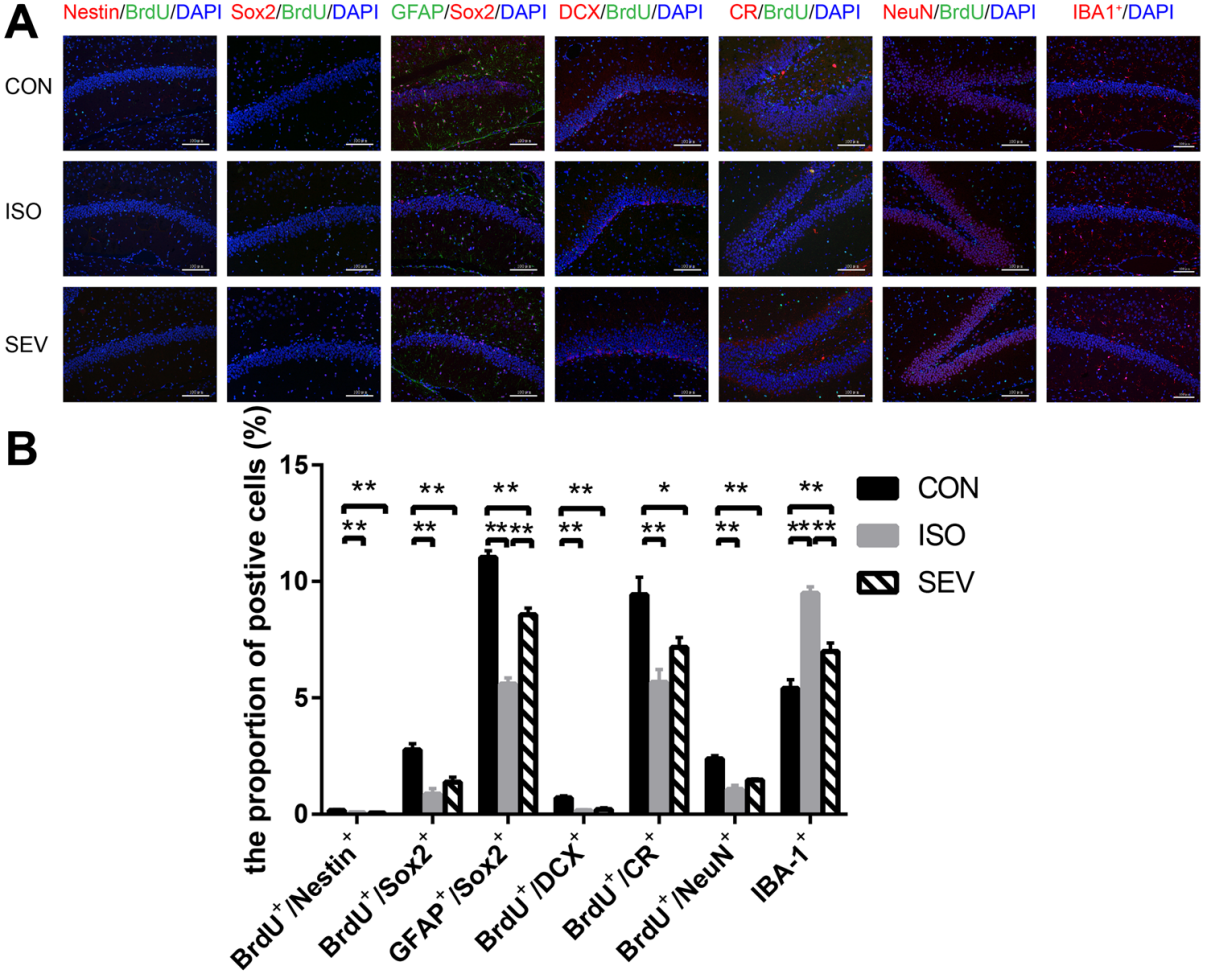
Neurodevelopmental markers for NSC in the hippocampus were stained 24 h after the MWM (P28). **A, B.** The results revealed that the levels of Nestin^+^/BrdU^+^, Sox2^+^/BrdU^+^, Sox2^+^/GFAP^+^, DCX^+^/BrdU^+^, CR^+^/BrdU^+^ and NeuN^+^/BrdU^+^ co-labeling decreased in the ISO (*p* = 0.006, for Nestin^+^/BrdU^+^; *p* = 0.000, for Sox2^+^/BrdU^+^; *p* = 0.000, for Sox2^+^/GFAP^+^; *p* = 0.000, for DCX^+^/BrdU^+^; *p* = 0.000, for CR^+^/BrdU^+^; *p* = 0.000, for NeuN^+^/BrdU^+^) or the SEV (*p* = 0.006, for Nestin^+^/BrdU^+^; *p* = 0.001, for Sox2^+^/BrdU^+^; *p* = 0.000, for Sox2^+^/GFAP^+^; *p* = 0.000, for DCX^+^/BrdU^+^; *p* = 0.016, for CR^+^/BrdU^+^; *p* = 0.000, for NeuN^+^/BrdU^+^) group compared with that in the CON group. Furthermore, the numbers of IBA-1^+^ cells increased in the ISO (*p* = 0.000) or the SEV (*p* = 0.005) group compared with that in the CON group. The level of Sox2^+^/GFAP^+^ co-labeling decreased in the ISO group compared with that in the SEV group (*p* = 0.000). The numbers of the IBA-1^+^ cells increased in the ISO group compared with that in the SEV group (*p* = 0.000). Data are mean ± SEM. **p < 0.01, *p < 0.05. Images were captured with 200 × magnification

**Fig. 9:**
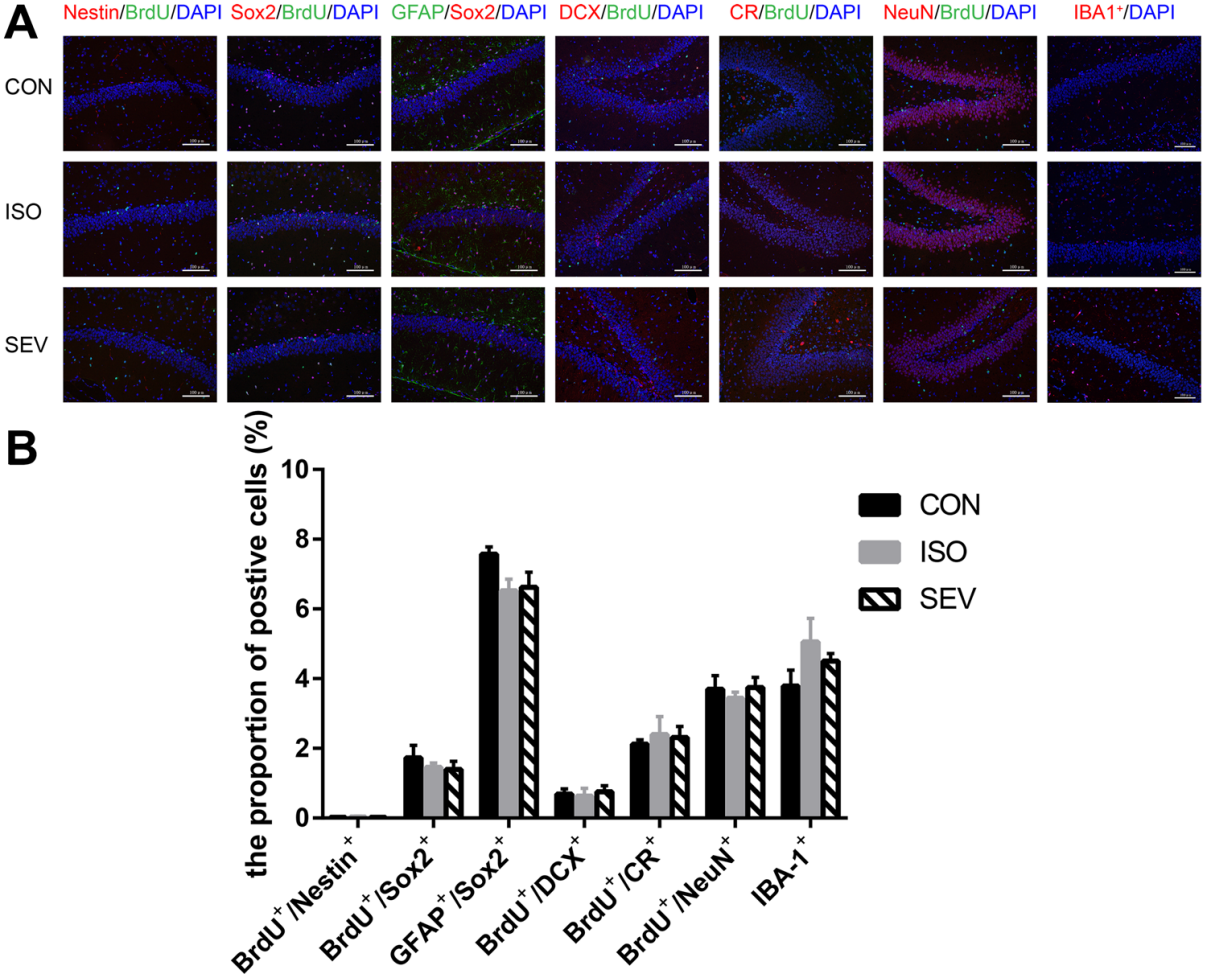
Neurodevelopmental markers for NSC in the hippocampus were stained 24 h after the second MWM (P56). **A, B.** The results revealed that all the co-labeling markers had no significant difference. Data are mean ± SEM. Images were captured with 200 × magnification

At P28, 24 h after the MWM, we observed reduced Nestin^+^/BrdU^+^, Sox2^+^/BrdU^+^, Sox2^+^/GFAP^+^, DCX^+^/BrdU^+^, CR^+^/BrdU^+^ and NeuN^+^/BrdU^+^ co-labeling in the ISO group (Fig. 8A,B, p = 0.006 for Nestin^+^/BrdU^+^; *p* = 0.000 for Sox2^+^/BrdU^+^; *p* = 0.000 for Sox2^+^/GFAP^+^; *p* = 0.000 for DCX^+^/BrdU^+^; *p* = 0.000 for CR^+^/BrdU^+^; *p* = 0.000 for NeuN^+^/BrdU^+^) and SEV group (Fig. 8A,B; *p* = 0.006 for Nestin^+^/BrdU^+^; *p* = 0.001 for Sox2^+^/BrdU^+^; *p* = 0.000 for Sox2^+^/GFAP^+^; *p* = 0.000 for DCX^+^/BrdU^+^; *p* = 0.016 for CR^+^/BrdU^+^; *p* = 0.000 for NeuN^+^/BrdU^+^) compared to the CON group. Furthermore, the number of IBA-1^+^ cells were significantly higher in the ISO group (Fig. 8A,B; *p* = 0.000) and SEV group (Fig. 8A,B; *p* = 0.005) compared to the CON group. Moreover, Sox2^+^/GFAP^+^ co-labeling was significantly lower in the ISO group compared to the SEV group (Fig. 8A,B, p = 0.000). The number of IBA-1^+^ cells were significantly higher in the ISO group compared to the SEV group (Fig. 8A,B; *p* = 0.000).. However, at P56, 24 h after the second MWM, we did not observe any differences in the co-labeling markers for all three groups (Fig. 9A,B).

## Discussion

### Isoflurane and Sevoflurane Induce Cognitive Impairment in Neonatal Rats

Our study showed that exposure to 1.1% isoflurane or 2% sevoflurane for 4 h impaired spatial memory in neonatal rats. The MWM escape latency was significantly longer in the ISO and SEV group rats compared to those in the CON group. Moreover, rats in the ISO group showed more persistent and prolonged escape latency than those in the SEV group. T maze task results showed that the percentage of correct selections (alternations) was significantly lower in the ISO and SEV group rats compared to the CON group rats on days 1 and 8. On days 7 and 10, the percentage of correct selections was significantly lower in the ISO group rats compared to the CON group rats, whereas, the differences between the SEV and CON group rats were not statistically significant. Overall, the results of the T maze tests showed that isoflurane and sevoflurane impaired working memory of the rats at 2 weeks. Furthermore, isoflurane induced more severe and prolonged neurotoxicity compared to sevoflurane at 2 weeks. The control rats from day 2 till 5 showed decreased percentage of correct alteration which is surprised but similar to that in a previous study (Mogensen et al. 2008; Schaefers and Winter 2011). The possible explanation could be that there were indications for behavioral asymmetries, since most rats showed a rightward direction of turning within the start arm (Castellano et al. 1987; LaHoste et al. 1988). Such biases may affect the outcome of experimental manipulations, be they behavioral or physiological (Andrade et al. 2001). It has been shown that the likelihood of asymmetry and its degree can be affected by time of testing (Schwarting and Borta 2005). Therefore, the performance could improve with time as seen after day 5.

The results of MWM and T maze tests of rats at 6 weeks after exposure to anesthetics was similar to the control group rats. This finding was consistent with previous reports (Stratmann et al. 2009b). This showed that cognitive function was completely restored in rats at 6 weeks after exposure to isoflurane or sevoflurane. A previous study reported that exposure of P7 rats to 1 MAC isoflurane for 4 h caused persistent deficits in spatial reference memory (Stratmann et al. 2009a, b). Another study reported that exposure to 1.5% isoflurane for 4 h caused cognitive impairment in P7 rats (Schaefer et al. 2019). Furthermore, cognitive function of P6 mice was significantly impaired after daily exposure to 3% isoflurane for 2 h over 3 consecutive days (Shen et al. 2013; Yu et al. 2020). Our results were consistent with the results from these studies. However, our results were contrary to findings from another study, which reported that cognitive impairment was induced at 6 weeks after isoflurane or sevoflurane exposure in neonatal animals (Zhu et al. 2010). We speculate that these differences might be due to variations in the age of rats, protocol for exposure to anesthetics, concentration of anesthetics, exposure time, or use of different animal strains.

### Isoflurane and Sevoflurane Inhibit Neurogenesis by Suppressing Proliferation and Differentiation of NSCs

Isoflurane anesthesia in rats altered postnatal hippocampal neurogenesis in an age-dependent manner (Erasso et al. 2013). The dentate gyrus restored normal numbers of GFP^+^-expressing granule cells in the Gli1-CreER::GFP bitransgenic mice on day 60 after a single, developmental exposure to 1.5% isoflurane for 6 h, thereby suggesting increased proliferation of GFP^+^ granule cells (Jiang et al. 2016). The proliferation of neuronal progenitor cells was significantly reduced for 5 days after administration of P7 rats with one minimum alveolar concentration (MAC) isoflurane for 4 h (Stratmann et al. 2009b). P14 mice and rats anesthetized with 1.7% isoflurane for 35 min daily over 4 successive days showed reduced hippocampal stem cell pool including radial glia-like stem cells and neurogenesis until 4 weeks after anesthesia (Zhu et al. 2010). Moreover, maternal exposure to 3.5% sevoflurane during the mid-gestational period inhibited fetal NSC proliferation via the Wnt/β-catenin pathway and impaired postnatal learning and memory function in rats (Wang et al. 2018). Exposure of P7 rats to 2.5% sevoflurane for 9 h inhibited proliferation of neural progenitor cells until 2 weeks after anesthesia (Liu et al. 2014).

It has been shown that drugs that act by enhance GABA_A_ receptors (isoflurane or sevoflurane) or block NMDA receptors (ketamine or nitrous oxide) induce neurotoxicity in immature rodent brain when administered during synaptogenesis. Neonatal exposure to ketamine in rats inhibits the proliferation and astrocytic differentiation of NSCs in the hippocampal dentate gyrus and impairs neurocognitive function in adulthood (Huang et al. 2021). Repeated neonatal ketamine exposure inhibits the proliferation and astrocytic differentiation of NSCs in the SVZ and induces olfactory cognitive dysfunction in the adulthood (Sha et al. 2021). Furthermore, ketamine exposure inhibits proliferation and neuronal differentiation of NSCs in the SVZ and SGZ of the hippocampus and leads to adult cognitive deficits (Li et al. 2019). Nitrous oxide is similar in nature to ketamine. Long-term impairments of neuronal development and synaptic communication could be caused when P7 rats were exposed to a sedative dose of midazolam followed by combined nitrous oxide and isoflurane anesthesia for 6 h (Dalla Massara et al. 2016). Recent research has raised concerns about possible neurotoxicity of nitrous oxide, particularly in the developing brain (Savage and Ma 2014; Shu et al. 2010). Neonatal exposure to nitrous oxide (75% N_2_O and 25% O_2_) in mice inhibits cell proliferation in developing brain (Rodier et al. 1986). Neurogenesis can be down-regulated after exposure to nitrous oxide (Covacu et al. 2006). However, the neurotoxic effects of nitrous oxide are not observed for the P5-6 rhesus monkeys (Zou et al. 2011).

Our results showed that isoflurane and sevoflurane inhibited the proliferation and differentiation of NSCs at P12 or P28. Moreover, the effects of isoflurane-induced neurodevelopmental toxicity were stronger than those of sevoflurane. These results were consistent with previous reports. Moreover, proliferation and differentiation of the NSCs in the isoflurane and sevoflurane groups were similar to the control group when analyzed at P56. Our study showed that the neurodevelopmental toxicity observed after exposure of P7 rats to the anesthetics for 4 h persisted for at least 3 weeks after anesthesia.

### Microglial Activationmediates in Vivo Neurotoxicity of Hippocampal NSCs

Hippocampal neurogenesis was inhibited and the numbers of microglia in the dentate gyrus were significantly increased after peripheral LPS injections in rodents (Belarbi et al. 2012; Dinel et al. 2014; Valero et al. 2014). However, LPS-induced inhibition of neurogenesis was abrogated by anti-inflammatory drugs (Monje et al. 2003). Systemic administration of minocycline in adult rats restored hippocampal neurogenesis, which was significantly reduced after intracortical injection of LPS for 28 days; moreover, a negative correlation was observed between the number of new neurons and the number of activated microglia (Ekdahl et al. 2003). Microglial activation was observed in multiple brain regions of male newborn piglets that were exposed to 2.0% isoflurane for 6 h (Broad et al. 2016). These findings suggested that anesthesia-induced neurodevelopmental toxicity was related to the release of inflammatory cytokines by activated microglia.

TNF-α secretion from the microglia aggravates neurotoxicity of hippocampal NSCs in co-culture experiments (Cacci et al. 2005; Iosif et al. 2008). Moreover, cytokines and chemokines released during neuroinflammation inhibited neurogenesis (Monje et al. 2003). However, microglia also release factors that promote proliferation and maintenance of the NSCs (Deierborg et al. 2010). IL-6 levels are increased when primary murine microglia are exposed to 2% isoflurane or 4.1% sevoflurane for 6 h (Wang et al. 2014; Zhang et al. 2013). Furthermore, pretreatment of primary microglia with 2% or 4% sevoflurane inhibited IL-4-induced M2 microglial activation and decreased the levels of IL-10, Arg1, and Ym1 (Pei et al. 2017). Previous studies also showed that inhaled anesthetics increase the levels of inflammatory cytokines, such as TNF-α, IL-6, and IL-1β in the brains of experimental mice (Shen et al. 2013) and induce neurotoxicity (Wu et al. 2012). However, sevoflurane post-conditioning can also exert anti-inflammatory and neuroprotective effects. For example, sevoflurane post-conditioning decreased infarct size, improved neurological deficit score, and reduced the levels of pro-inflammatory cytokines, such as TNF-α and IL-6 (Ye et al. 2015).

Our results showed increased number of IBA-1^+^ cells in the brain after isoflurane and sevoflurane exposure. Moreover, the levels of CD11b, IL-6, and TNF-α were increased in the hippocampus of rats exposed to isoflurane and sevoflurane. This demonstrated that isoflurane and sevoflurane induced neurodevelopmental toxicity by activating microglia and up-regulating the release of inflammatory cytokines.

### Isoflurane and Sevoflurane Inhibit Proliferation and Differentiation of NSCs by Suppressing the VEGFR2 Signaling Pathway

VEGFR2 signaling pathway promotes proliferation, differentiation, and migration of neural stem cells (Jin et al. 2002; Mani et al. 2010). Our results showed that VEGFR2 activity was significantly decreased (reduced levels of p-VEGFR2 and low ratio of p-VEGFR2/VEGFR2) in the hippocampus of rats exposed to isoflurane and sevoflurane. This suggested that anesthesia-induced neurodevelopmental toxicity was also associated with reduced VEGFR2 activity in addition to increased microglial activation.

There is a limitation of our study. Isoflurane or sevoflurane induced “brain damage” through the interaction between microglia and NSCs and the level of VEGFR2 phosphorylation may be just an association rather the “real” mechanism. Therefore, pharmacological intervention (such as PLX3397 for microglia elimination) or conditional knockout rodents should be considered in future study to verify the mechanism of anesthetics-induced neurodevelopment toxicity.

## Conclusions

Our results demonstrated that exposure to isoflurane and sevoflurane caused learning and memory impairments in neonatal rats because of reduced proliferation and differentiation of NSCs, up-regulated neuroinflammation including activated microglia and increased levels of pro-inflammatory cytokines such as IL-6 and TNF-α, and decreased ratio of p-VEGFR2/VEGFR2 in the brain. These adverse effects persisted until 3 weeks after anesthetics exposure. However, we also observed cognitive function was completely restored 6 weeks after anesthetics exposure in the rats. The results of our study may shed some light on the potential role of microglia and VEGFR2 in anesthetics-induced neurotoxicity. It may be the beneficial effects of inhibiting microglia activation or promoting the VEGFR2 phosphorylation in anesthetics-induced cognitive dysfunction. However, further studies are needed to establish the cause-effect relationships among these factors.
